# Lactic Acid Fermentation Ameliorates Intrinsic Toxicants in *Brassica campestris* L. Leaves Harvested at Different Growth Stages

**DOI:** 10.3390/foods13121826

**Published:** 2024-06-11

**Authors:** Muhammad Younis, Saeed Akhtar, Tariq Ismail, Muhammad Qamar, Dur-e-shahwar Sattar, Wisha Saeed, Mohammad S. Mubarak, Elena Bartkiene, João Miguel Rocha

**Affiliations:** 1 Department of Food Science and Technology, Faculty of Food Science and Nutrition, Bahauddin Zakariya University, Multan 60800, Pakistan; younismian0@gmail.com (M.Y.); tariqismail@bzu.edu.pk (T.I.); muhammadqamar@bzu.edu.pk (M.Q.); dsattar@bzu.edu.pk (D.-e.-s.S.); wishasaeed1@gmail.com (W.S.); 2 Department of Chemistry, The University of Jordan, Amman 11942, Jordan; mmubarak@ju.edu.jo; 3 Institute of Animal Rearing Technologies, Lithuanian University of Health Sciences, Tilzes Str. 18, LT-47181 Kaunas, Lithuania; elena.bartkiene@lsmu.lt; 4 Department of Food Safety and Quality, Lithuanian University of Health Sciences, Tilzes Str. 18, LT-47181 Kaunas, Lithuania; 5 CBQF—Centro de Biotecnologia e Química Fina—Laboratório Associado, Escola Superior de Biotecnologia, Universidade Católica Portuguesa, Rua Diogo Botelho 1327, 4169-005 Porto, Portugal; 6 LEPABE—Laboratory for Process Engineering, Environment, Biotechnology and Energy, Faculty of Engineering, University of Porto, Rua Dr. Roberto Frias, s/n, 4200-465 Porto, Portugal; 7 ALiCE—Associate Laboratory in Chemical Engineering, Faculty of Engineering, University of Porto, Rua Dr. Roberto Frias, s/n, 4200-465 Porto, Portugal

**Keywords:** phytochemicals, anti-nutrients, probiotic microorganism, *Lactiplantibacillus plantarum*, antioxidant potential

## Abstract

*Brassica campestris* (syn. *Brassica rapa*) is often known as mustard and is grown worldwide owing to its health-promoting characteristics associated with the presence of nutrients and phytochemicals. Along with the nutritional components, *B. campestris* also contains anti-nutrients (phytates, oxalates, tannins, alkaloids, saponins) that can cause adverse severe health effects to consumers, including rashes, nausea, headaches, bloating and nutritional deficiencies. In the present study, heating (blanching) and fermentation (*Lactiplantibacillus plantarum*) treatments were applied to reduce the load of the anti-nutrients of *B. campestris* leaves harvested at three different growth stages: the first stage (fourth week), the second stage (sixth week) and the third stage (eighth week). Results revealed that fermentation treatment using *Lp. plantarum* increases the ash (5.4 to 6%), protein (9 to 10.4%) and fiber (9.6 to 10.7%) contents, whereas moisture (0.91 to 0.82%), fat (9.9 to 9.1%) and carbohydrate (64.5 to 64.2%) contents decreased among *B. campestris* samples, and the trend was similar for all three stages. Blanching and fermentation lead to the reduction in phytates (46, 42%), saponins (34, 49%), tannins (1, 10%), oxalates (15, 7%) and alkaloids (10, 6%), separately as compared to raw samples of *B. campestris* leaves. In contrast, fermentation had no considerable effect on phytochemical contents (total phenolic and total flavonoids) and antioxidant potential (DPPH and FRAP). The action of blanching followed by fermentation caused more decline in the aforementioned toxicants load as compared to blanching or fermentation alone. Structural modifications in blanching and the biochemical conversions in fermentation lead to enhanced stability of nutrients and antioxidant potential. Taken together, these findings suggest blanching followed by fermentation treatments as a reliable, cost-effective and safer approach to curtail the anti-nutrient load without affecting the proximate composition, phytochemical attributes and antioxidant activity.

## 1. Introduction

*Brassica campestris* L. (syn. *Brassica rapa* L.) is the earliest cultivated plant, dating back to 5000 years, and is predominantly consumed as a green leafy vegetable in the South Asian Subcontinent regions [1]. *B. campestris* is commonly known as mustard, frequently cultivated worldwide as a food source [2,3]. Consumption of *B. campestris* is preferred in various parts of the world owing to its health-promoting properties due to the presence of nutrients and phytochemicals, including carotenoids, B-vitamins, minerals (calcium, iron, etc.), dietary fibers, soluble sugars and fatty acids [4,5]. *B. campestris* also contains notable amounts of anti-nutrients, and these are simple amines, peptides, alkaloids (nitrates, nitrites, oxalates, phytates, tannins), glycosides and phenolic compounds that contribute to the natural defense system of the plants against a range of biological threats. However, they negatively influence the bioavailability of nutrients, such as minerals, vitamins and proteins in humans [6,7]. These compounds could be of natural as well as synthetic origin and could cause conditions including rashes, nausea, headaches, bloating and nutritional deficiencies [6,8].

Abundant literature dealing with the technologies is available, including different chemical treatments, cooking, heating, blanching, soaking, fermentation, germination (sprouting) and radiation, among others, that are employed to reduce natural toxicants [9]. Although these techniques are slightly effective in reducing the anti-nutrients, they also cause some quality losses, such as the loss of water-soluble vitamins. The selection of treatment to reduce the anti-nutrients, along with quality retention or quality impairment, is a matter of great concern amongst scientists working in the domain of food science and technology.

Fermentation is regarded as one of the oldest and most successful methods for extending the shelf-life of foods without refrigeration since it enhances the nutritional value of the food products while also adding distinctive flavor and texture [10]. The bacterial species *Lactiplantibacillus plantarum* is extensively used in the food industry as a probiotic microorganism and is a microbial starter to produce functional, traditional and novel foods and beverages with improved nutritional and technological features. Regarding yield, maximum potential and viability, *Lp. plantarum* strains possess great ability to survive in the gastrointestinal tract (GI) and adhere to its epithelial cells and, most importantly, are safe strains for animals and humans [11]. By utilizing various microbiota and improving the approach or techniques to reduce the toxicant level in the chosen vegetable crop, the current study was largely designed to lessen the toxicological burden of *B. campestris* vegetables. In the present investigation, intrinsic anti-nutrients of *B. campestris* leaves harvested at three different growth stages were determined and reduced to minimum levels, adopting fermentation using *Lp. plantarum* active culture. The application of the findings of the present study at the industrial level will go a long way to enhance the processing of selected vegetable crops with improved health features.

## 2. Materials and Methods

### 2.1. Procurement of B. campestris Leaf Samples

*Brassica campestris* L. (syn. *Brassica rapa* L.) was grown in the agricultural fields of the Faculty of Food Science and Nutrition (FFSN) at Bahauddin Zakariya University (BZU), Multan, Pakistan. Botanists at the Department of Botany, BZU, Multan, Pakistan, identified and authenticated the plant. A reference sample for traceability purposes was kept in the Department of Food Science and Technology, BZU, Multan, Pakistan. This was followed by destalking, sorting, washing and draining of the leaves of the selected plant samples.

### 2.2. Morphological Attributes of B. campestris Plant at Different Growth Stages

*B. campestris* was grown organically on 8 October 2022, and its root length, stem length, leaf length and width, plant height and leaf count were measured at the 1st stage (4th week), 2nd stage (6th week) and 3rd stage (8th week) of plant growth. Plant height, root size, stem length and leaf length were measured using a ruler, while leaf number was manually counted. These morphological attributes of the plant were measured to understand the length of the plant with maximum leaf count at each stage having maximum levels of anti-nutrients [12].

### 2.3. Sample Preparation of B. campestris Leaves

The fresh leaves of *B. campestris* (2 Kg) were plucked/destalked at the 1st, 2nd and 3rd stages of plant growth. The leaves were sorted, cleaned, washed and drained for further chemical analysis. Leaves at each stage were again categorized as RBC (raw *B. campestris*) (controlled sample), BBC (blanched *B. campestris*) (steam blanched in a water bath at 90 °C for 3 min) and FBC (fermented *B. campestris*) (fermentation through *Lp. plantarum*). Fermentation was carried out by taking fresh leaves of *B. campestris* from each stage [13].

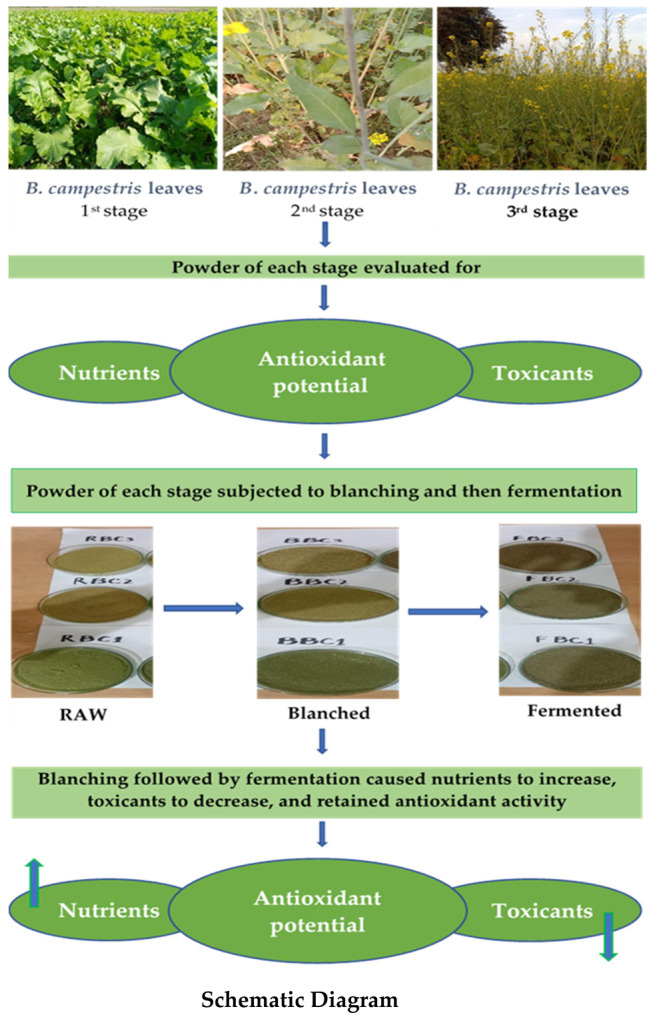



### 2.4. Fermentation of B. campestris Fresh Leaves Harvested at Different Growth Stages

The *B. campestris* leaves harvested at three different growth stages underwent the submerged fermentation process using *Lp. plantarum* v. 299 strains. The activation of the strains was performed in MRS broth by incubating (Memmert BE500 incubator, Malente, Germany) the culture at 32 °C for 48 h. The biomass was centrifuged (Model Hermle, Z326-K, Malente, Germany) at 4000 rpm for 5 min at 4 °C and suspended in 0.9% saline water solution to preserve a bacterial cell count of 10^7^ CFU/mL. The leaves of *B. campestris* were chopped into small pieces and steam blanched at 90 °C for 3 min and then cooled down to room temperature. A 100 g sample of chopped, blanched leaves was placed in a glass vessel that had been sterilized, and 200 mL of distilled water combined with 2 g of NaCl and 2 g of maltodextrin was added. In order to initiate the fermentation process by intermittent shaking, 0.2 mL of the previously prepared *Lp. plantarum* inoculum suspension was also added [14,15,16]. The fermentation process was carried out in an incubator (Memmert Incubator Beschickung/loading-Modell 100–800, Malente, Germany) for 24 to 48 h at 32 °C to attain the sample pH between 4.0 and 4.1. The fermentation process was monitored by controlling the physical conditions, i.e., temperature, pH and titratable acidity of the sample after every 6 h period.

### 2.5. Determination of pH and Acidity during the Fermentation Process

After every 6 h, 5 mL of the fermentation solution was aseptically removed to measure the pH with a pH meter (Model: ST3100 OHAUS corporation, Parsippany, NJ, USA) [13]. Acidity was calculated by diluting 10 mL of fermentation solution with 50 mL of water and titrating to pH 8.00 with 0.1 N NaOH. The quantity of titrant used to neutralize the acid was noted. The purpose of measuring the pH and titratable acidity was to stop the fermentation process with the required characteristics of the sample, and the following formula (Equation (1)) was used to determine the solution’s titratable acidity [17]:

(1) $$Titratable\;acidity\;\left(\%\right)=\frac{volume\;of\;titrant\times normality\;of\;titrant\times equivalent\;weight\;of\;acid}{volume\;of\;sample\times 1000}\times 100$$

### 2.6. Dehydration of Raw, Blanched and Fermented B. campestris Leaves

RBC, BBC and FBC leaf samples of all stages were spread over the trays and placed in the cabinet dryer to dehydrate the treated leaves at 40 °C (Pamico Technologies Faisalabad, Punjab, Pakistan) for comparative toxicological and nutritional assessments. Treated leaves at each stage were collected separately and blended independently using a laboratory blender (Pamico Technologies Faisalabad, Pakistan) for powder production. The powder was packed in airtight plastic containers, labeled and stored at room temperature for further analysis [18,19].

### 2.7. Chlorophyll a and b, Total Chlorophyll and Anthocyanin Content Analysis

A clean pestle mortar was used to gently blend 1 g of homogenized leaf powder material with 10 mL of 80% acetone and 0.5 g of magnesium carbonate (MgCO_3_) powder; the blend was placed in a 40 °C refrigerator for 4 h. The sample was centrifuged for five minutes at 5000 rpm, and the supernatant was poured into test tubes. Absorbance of the solution was determined at 645 and 663 nm wavelengths against the solvent using a spectrophotometer (V-3000; VWR, Darmstadt, Germany); acetone (80%) was employed as a blank. The following equations (Equations (2)–(4)) were used to measure chlorophyll a and b and total chlorophyll contents in *B. campestris* leaf samples [20]:

(2) Chl a=11.75×Absorbance at 663−2.35×Absorbance at 645

(3) Chl b=18.61×Absorbance at 645−3.96×Absorbance at 663

(4) Total chlorophyll=chl a+chl b

where Chl a and Chl b are chlorophyll a and chlorophyll b [20].

Total monomeric anthocyanins from *B. campestris* leaf powders were determined using the Association of Official Analytical Chemists (AOAC) guidelines by preparing the aqueous buffer solutions of 0.025 M potassium chloride at pH 1 and 0.4 M sodium acetate at pH 4.5, with the pH adjusted with concentrated HCl [21]. For this, 200 µL of each sample extract and 1.8 mL of each buffer were placed in separate test tubes and mixed. After equilibrating at room temperature in the dark for 15 min, the absorbance was measured at 510 and 700 nm using a UV–visible spectrophotometer (V-3000; VWR, Darmstadt, Germany) with distilled water as the blank [22]. The following formula (Equation (5)) was used to estimate the monomeric anthocyanin concentration in *B. campestris* leaf powder samples:

(5) $$Total\;anthocyanin\left(mg\;cyanidin-3-glucose/L\right)=\frac{A\times MW\times DF\times 1000}{\varepsilon \times 1}$$

where A is [(pH 1: A 510 nm-A 700 nm) − (pH 4.5: A 510 nm-A 700 nm)], DF is the dilution factor (10 × following the procedure given), MW is the molecular weight (449.38 g mol^−1^), ε is the molar extinction coefficient (ε = 26,900 M^−1^ cm^−1^), and 1 is the path length in cm.

### 2.8. Proximate Analysis of the Samples Harvested at Different Growth Stages

Moisture, total ash, crude fat, crude protein, crude fiber and nitrogen-free extract (NFE) contents present in raw, blanched and fermented dried *B. campestris* samples of all stages were analyzed following the AOAC guidelines [21]. The moisture contents of the samples were measured by the hot air oven drying method at 105 °C, whereas the total ash contents were determined by incinerating the samples in a muffle furnace at 550 °C for 4 h. Crude protein contents were determined by the micro Kjeldahl method using 6.25 as the conversion factor [23]. In contrast, crude fat was determined using the Soxhlet extractor with *n*-hexane as a solvent. The amount of crude fiber was evaluated using the acid and base digestion method at Fiber Tech [23,24] by employing the following equation (Equation (6)) to quantify the amount of readily available carbohydrates or nitrogen-free extracts (NFEs) in the samples:

(6) $$NFE\left(\%\right)=100-(Moisture\%+Ash\%+Fiber\%+Protein\%+Fat\%)$$

The gross food energy (Kcal/100 g) was estimated through water conversion factors (Equation (7)) by multiplying the crude protein, crude fat and total carbohydrate values by 4, 9 and 4, respectively [24,25]:

(7) Energy (Kcal/100 g)=(4×crude Protein)+(9×crude Fat)+(4×carbohydrates)

### 2.9. Color Tonality of B. campestris Fresh Leaves and Powders

The color tones of fresh leaves and powder samples of *B. campestris* were assessed using a colorimeter with illuminant D65, A, C, D50 (spectrophotometer, YS3010, 3NH Technology Co. Ltd., Shenzhen, China) that was calibrated using a reference white plate and black grove. For each sample, three measurements of each coordinate L* (lightness/darkness), a* (redness/greenness) and b* (yellowness/blueness) were measured according to published procedures [26].

### 2.10. DPPH Radical Scavenging Activity

The DPPH (2,2-diphenyl-1-picrylhydrazyl) method was used to estimate the radical scavenging activity (RSA) of the crude extracts of *B. campestris* leaf samples [27]. Briefly, in a nutshell, 2 mL of DPPH (0.1 mM) solution was added to 2 mL of extract solution (1–100 g/mL) in acetone, ethanol, *n*-hexane and methanol. The mixtures were set aside in a dark place for 30 min, then the absorbance at λmax 517 nm was determined using a UV-Vis spectrophotometer (V-3000; VWR, Darmstadt, Germany) in comparison to a control mixture of DPPH and methanol in the same amount. The following equation (Equation (8)) was used to estimate the % DPPH radical scavenging activity (% DPPH RSA):

(8) $$\%\;DPPH\;inhibition=\frac{absorbance\;of\;the\;control-absorbance\;of\;the\;test\;extracts}{absorbance\;of\;the\;control}\times 100$$

### 2.11. Total Phenolic Content (TPC)

The Folin–Ciocalteu method, reported by Singleton and coworkers [28] and modified by Dewanto et al. (2002) [29], was used to quantify the total phenolic contents (TPC) of powder extracts of *B. campestris* leaves produced in acetone, ethanol, *n*-hexane and methanol. Briefly, 0.5 mL of distilled water, 125 µL of the Folin–Ciocalteu reagent and 125 µL of the appropriately diluted extract of each sample were mixed in separate test tubes. Afterwards, each test tube mixture was equilibrated for 6 min, 1.25 mL of 7% Na_2_CO_3_ solution was added, and the mixtures’ final volumes were adjusted to 3 mL. Then, test tubes were let to stand for 90 min, while the absorption was measured at 760 nm using a UV-Vis spectrophotometer (Specord 200 Plus, analytikjena, Thuringia, Germany) with water used as a blank. The amount of total phenolics was presented as gallic acid equivalents (GAE, mg gallic acid/g sample) using the calibration curve for gallic acid [30].

### 2.12. Ferric Reducing Antioxidant Power (FRAP) Assay

The method of Benzie and Strain [31] was used to evaluate the ferric-reducing capacities of the powder leaf extracts of *B. campestris* produced in acetone, ethanol, *n*-hexane and methanol. In a nutshell, aliquots of the samples (100 µL) were combined with the ferric reducing antioxidant power (FRAP) reagent (3 mL) and, after 6 min of incubation at 37 °C in a water bath, the absorbance at 593 nm was measured on a UV-Vis spectrophotometer (V-3000; VWR, Darmstadt, Germany). Results were expressed as μmol of FeSO_4_ equivalents per gram defatted samples [32].

### 2.13. Total Flavonoid Content (TFC)

The method reported by Xu and Chang [33] was used, with slight modification, to measure the total flavonoid content (TFC) of powder *B. campestris* leaf extracts. For such a purpose, 0.3 mL of 5% NaNO_2_ solution and 1 mL of suitably diluted sample extract were combined in a test tube. After five minutes of incubation, 0.3 mL of a 10% AlCl_3_ solution was added, followed by the addition of 2 mL of a 1 M NaOH solution. The absorbance of each sample was measured at 510 nm using a UV-Vis spectrophotometer (V-3000; VWR, Darmstadt, Germany) [32].

### 2.14. Estimation of Phytates (Phytic Acid)

In this method, a 0.06 g sample from each treatment of the *B. campestris* leaf powder was extracted with 10 mL of 0.2 N HCl solution. Following the extraction, aliquots of 0.5 mL of each extract were placed into 10 mL test tubes and enclosed with stoppers. Then, 1 mL of ferric solution (0.2 g of NH_4_Fe(SO_4_)_2_·12H_2_O in 1000 mL of 2 N HCl) and 2 mL of distilled water were added to each test tube. The tubes were heated in a boiling water bath for 30 min. After cooling in ice water for 15 min, the tubes were allowed to adjust to room temperature. This was followed by the addition of 0.25 mL of 15% (*w*/*v*) potassium thiocyanate (KSCN) solution (15 g of KSCN in distilled water to make the volume 100 mL), and the contents were mixed. Absorbance (OD, optical density) was recorded at 480 nm after 25–30 min using a UV-Vis spectrophotometer (V-3000; VWR, Darmstadt, Germany) [34,35,36].

### 2.15. Determination of Tannin Contents

Accurately weighed 0.5 g samples of the powdered *B. campestris* leaves and 75 mL of distilled water were transferred to 250 mL conical flasks. The samples were gently heated to boiling for 30 min and then centrifuged for 20 min at 2000 rpm. The supernatant was collected in a 100 mL volumetric flask, and the volume was made up to the mark with distilled water. Then, 1 mL of the sample extract and 75 mL of distilled water were transferred to a 100 mL volumetric flask. Following this, 5 mL of Folin–Denis reagent and 10 mL of sodium carbonate solution were added, and the whole mixture was made up to 100 mL with distilled water and shaken well. The absorbance of the samples was measured using a UV-Vis spectrophotometer (V-3000; VWR, Darmstadt, Germany) at 700 nm after a 30-min incubation [37].

### 2.16. Total Saponin Content

The saponin contents of the samples were determined by the method of Dini et al. [38] with slight modifications. Briefly, aliquots of 0.1 mL samples were mixed with 1 mL of 72% sulfuric acid and 0.1 mL of 8% freshly prepared vanillin solution in ethanol. The mixtures were placed in a 60 °C water bath for 20 min and then cooled in ice-cold water. The absorbances of samples were measured at 544 nm with the aid of a UV-Vis spectrophotometer (Specord 200 Plus, analytikjena, Thuringia, Germany), and saponin content was calculated from a standard curve constructed with purified soy saponin standard [32].

### 2.17. Oxalate Contents

In this test, 0.5 g of plant material was weighed in an electric weighing balance, transferred to 30 mL of 0.25 N HCl (2.1 mL concentrated HCl in 100 mL of distilled water) and boiled in a water bath for 15 min. The total extract volume was made to 50 mL by adding already prepared 0.25 N HCl. Then, 1 mL of plant extract was added with 5 mL of 2 N H_2_SO_4_ (5.44 mL H_2_SO_4_ in 100 mL distilled water to make 2 N H_2_SO_4_) and 2 mL of 0.003 M KMnO_4_ (0.048 g KMnO_4_ in 100 mL solution with distilled water). The absorbance of the mixture was measured at 519 nm with the aid of a UV-Vis spectrophotometer (V-3000; VWR, Darmstadt, Germany) after being incubated at 37 °C for 10 min. A standard solution of oxalic acid was prepared by dissolving 100 mg of oxalic acid (C_2_H_2_O_4_·2H_2_O) in distilled water to make a 100 mL solution. From the standard oxalic acid solution, 0.1, 0.2, 0.3 and up to 1 mg/mL concentration solutions were prepared by proper standard dilutions. Furthermore, a series of standard solutions of oxalic acid containing 0.1, 0.2, 0.3 and up to 1 mg/mL concentration were mixed with 5 mL of 2 N H_2_SO_4_ and 2 mL of 0.003 M KMnO_4_ in separate test tubes and incubated at 37 °C for 10 min. A blank solution was prepared by adding 5 mL of 2 N H_2_SO_4_ and 2 mL of 0.003 M KMnO_4_ simultaneously with the solution mixture and incubated at 37 °C for 10 min [39].

### 2.18. Determination of the Alkaloid Contents

For this test, the *B. campestris* leaf powder extract residue was partially dissolved in 2 N HCl before being filtered. One milliliter of this solution was added to a separatory funnel that was rinsed three times with 10 mL of chloroform. The pH of this mixture was brought to a neutral level with 0.1 N NaOH. Then, 5 mL of phosphate buffer solution (pH 4.7) and 5 mL of bromocresol green solution (BCG) were added to the mixture. The mixture was vigorously stirred to extract the complex with 1, 2, 3, and 4 mL of chloroform. The extract was then collected in a 10 mL volumetric flask and diluted with chloroform. The absorbance of this mixture was measured at 470 nm with a UV-Vis spectrophotometer (V-3000; VWR, Darmstadt, Germany) and compared to a blank made as described above but without atropine. To quantify the absorbance, accurately measured aliquots of the atropine standard solution (0.4, 0.6, 0.8, 1.0 and 1.2 mL) were added to various separatory funnels together with 5 mL of phosphate buffer and 5 mL of BCG solution [40].

### 2.19. Statistical Analysis

All determinations were conducted in triplicate, and data were subjected to completely randomized design (CRD) with one-way analysis of variance (ANOVA). Statistical analysis was performed with the aid of different tests for statistically significant differences between treatments using Statistix 8.1. (2003) analytical software (Tallahassee, FL, USA); differences were considered significant at *p* ≤ 0.05.

## 3. Results

### 3.1. Morphological Attribute of B. campestris

The morphological parameters of *B. campestris* were recorded in the fourth, sixth and eighth weeks of plant growth. Shown in Table 1 are the attributes of *B. campestris* harvested at different growth stages. Results for the morphological attributes indicated statistically significant differences for each parameter as the plants become more mature and develop with time. Results showed that the plant’s roots were 7.22 inches in size at the end of the fourth week of growth and steadily grew to 10.04 and then 11.09 inches by the end of the sixth and eighth weeks, respectively. Similarly, the plant’s stem measured 12.10 inches at the end of the fourth week and increased to 74.14 inches after eight weeks of growth, showing that the stem’s size rose quickly with time. As the plant’s growth period lengthened, the length and width of its leaves increased; however, the range in leaf length was greater than that in leaf width. The plant’s stem and root sizes have an impact on the overall length of the plant as the height of the plant increases along with the size of the root and stem. Similarly, as the growth period and maturity stage of plants expand, the number of leaves on each plant also increases to the maximum because they need more chlorophyll and a larger surface area for the absorption of additional light to produce more energy.

### 3.2. pH and Acidity Changes during the Fermentation of B. campestris Leaves

**Figure 1:**
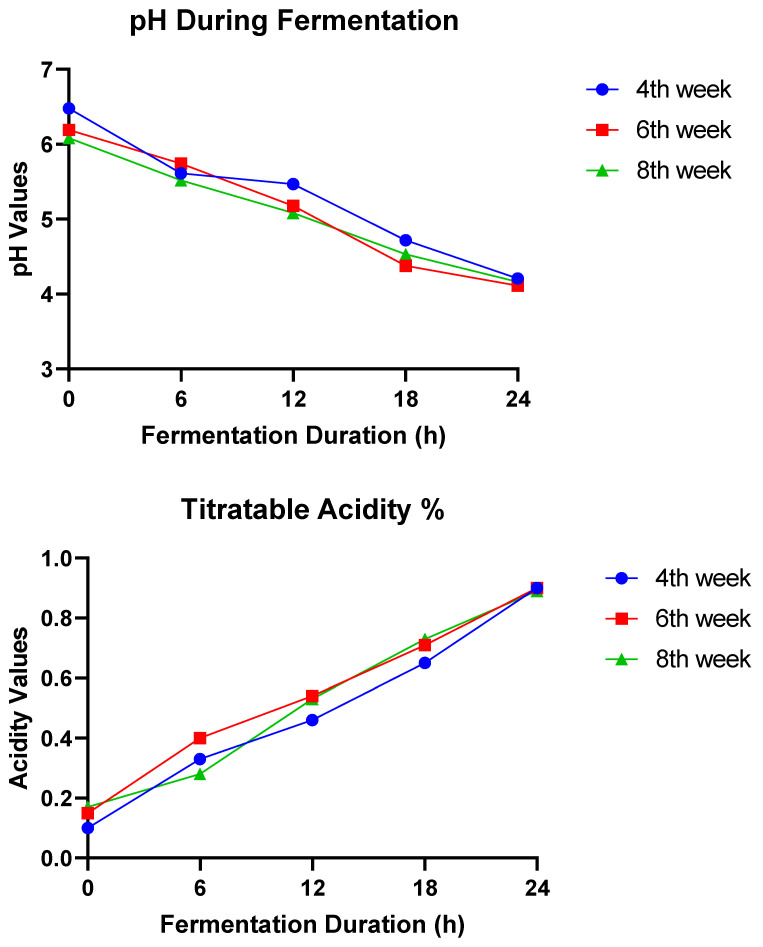
pH and acidity changes (mean ± standard deviation) during the fermentation of *B. campestris* leaves.

Shown in Figure 1 are the results of our study of the pH and acidity changes during the fermentation of *B. campestris* leaves. Our findings provide valuable insight into the pH and acidity variations that took place during the fermentation process of *B. campestris*. When *B. campestris* fresh leaves obtained at three distinct phases of development were fermented for 24 h, a decrease in pH values ranging from 6.48 to 4.11 (Figure 1) was observed. Results revealed that the pH of the samples gradually dropped as the fermentation time increased. Similarly, the titratable acidity of the fermentation samples gradually increased during the 24-h fermentation period of *B. campestris* leaves, ranging from 0.1 to 0.9.

### 3.3. Chlorophyll a and b, Total Chlorophyll and Anthocyanin Contents in B. campestris Leaves

Findings from this investigation showed that the fermented samples of *B. campestris* leaf powders have a maximum chlorophyll content (32.81–33.06) as compared to the raw and blanched treatments (Table 2). Similarly, fermentation could not cause a detrimental change in chlorophyll b contents (51.46 to 52.87) in comparison with raw and blanched samples, recorded to have intermediate levels. When comparing the treatments (raw, blanched and fermented) for total chlorophyll contents, results showed that fermented *B. campestris* stage 2 (FBC2) has the highest total chlorophyll contents (85.86), while blanched *B. campestris* stage 3 (BBC3) has the lowest total chlorophyll contents (77.04). The anthocyanin contents of *B. campestris* leaf (BcL) powder increased from stage 1 (134.33) to stage 2 (159.50), but a slight decrease was observed at stage 3 (158.85) as compared to stage 2 (Table 2). Results also indicated that blanching and fermentation cause a significant reduction in anthocyanin contents in the analyzed samples as compared to raw.

### 3.4. Proximate Composition of B. campestris

Table 3 shows our results of the proximate composition of *B. campestris* harvested at different growth stages. Findings showed that the moisture, ash and crude fat contents increase with the maturation stages, whereas varying impacts were recorded for crude protein and fiber. The *B. campestris*’ crude protein content peaked at the second stage of growth but fell as the length of growth increased. The estimated composition of *B. campestris* at varying growth stages was also significantly affected by blanching and fermentation. In this context, fermentation increased the ash, protein and fiber contents, whereas moisture and fat contents decreased among *B. campestris* samples, and the trend was similar for all three stages. On the other hand, moisture, ash and fiber contents decreased, while fat and protein contents increased upon exposure to blanching. Moreover, our results indicated that leaf samples of all stages of *B. campestris* are found to contain ash contents between 5.34 and 8.52%; the lowest was in the BBC1 and the highest in the FBC3 treatment, suggesting a negative impact of blanching and a positive one of fermentation. Likewise, the fat contents were recorded among all-stage leaf samples between 9.16 and 12.01%, with the amount being the lowest in the RBC2 and the highest in the BBC3 treatment, indicating that blanching treatment had a positive impact on the fat contents, while fermentation negatively affected the fat contents.

Furthermore, a positive impact of fermentation and blanching on the protein contents was observed; however, the increase was more pronounced for fermentation. The crude fiber contents of *B. campestris* ranged from 9.19 to 10.71%, showing that the growing period of the plant, as well as the blanching and fermentation treatments, had no notable effect on the crude fiber contents. In contrast, concentrations of the nitrogen-free extract (NFE) of *B. campestris* samples ranged from 57.46% in BBC3 leaf samples to 64.48% in BBC1 leaves. With the increased growth season and the application of fermentation and blanching treatments, NFE was greatly affected and reduced. The amount of protein, fat and carbohydrates in the samples of *B. campestris* leaves affected the gross energy they contain. The highest energy yield observed was in the range of 383.88 to 387.19 kcal/100 g in the different treatments of first, second and third growth stage harvested leaves, while the lowest energy (156.88 kcal/100 g) was yielded by RBC2 treatment.

### 3.5. Color Tonality of B. campestris Leaves

Findings from the study of the color tonality of the leaves (Table 4) revealed that the colors of the leaves of *B. campestris* vary as they develop and when various treatments are applied. The mean L* values of the fresh leaves at the first, second and third development stages in the FrBC1, FrBC2 and FrBC3 treatments were 54.80, 53.52 and 54.16, respectively, showing a moderate level of lightness (Table 4). This suggests that the young leaves are not excessively light or dark at any stage. Regarding the lightness of *B. campestris* leaf powders, the RBC3 treatment exhibited the highest mean L* value (66.65) compared to the FBC3 treatment, which showed the lowest mean L* value (51.88). Additionally, findings for the lightness of raw samples revealed that as the leaves expand to the third growing stage, they become marginally brighter. The a* values showed the degree of the green–red color component in the leaves; negative values denote a primarily green color, while positive values denote a shift toward red tones. The a* values are continuously negative across all treatments and growth phases, showing that the leaves of the *B. campestris* plant maintain a significant green color component, which is characteristic of healthy and chlorophyll-rich foliage (Table 4). Similarly, fresh leaves from all growth phases displayed the highest negative a* values, showing that green leaves have greater chlorophyll contents and maintain their green tone throughout all developmental stages. On the other hand, the negative b* values indicate a bluish tint and positive values denote a yellowish tint, describing the degree of the blue–yellow color component in the leaves. The b* readings consistently displayed a positive trend throughout treatments and growth stages, suggesting that *B. campestris* leaves typically have a yellowish tinge. Larger (more positive) b* values show that the leaves have a more pronounced yellow color when treatments at the same growth stage are compared. The fresh leaves of FrBC2 from the second growth stage exhibited the lowest mean b* value of 16.36, indicating that the new leaves from the second growth stage might contain some blueish taint.

### 3.6. Antioxidants Potential of the B. campestris Leaf Powder Extracts

Displayed in Table 5 are our results of the total phenolic content (TPC) of the acetone leaf extracts of *B. campestris* throughout the various samples and harvesting stages. Our findings showed that the TPC ranges from roughly 77.40 in BBC3 treatment to 246.78 mg GAE/g in FBC2, whereas the mean TPC values of the ethanol extracts ranged from 338.82 to 388.28 mg GAE/g. *n*-Hexane extracts exhibited the highest TPC value (367.71 mg GAE/g) in the FBC2 treatment, while the BBC1 treatment displayed the lowest mean TPC value of 93.88 mg GAE/g. Similarly, the TPC values of the methanol extracts ranged from 279.66 to 387.35 mg GAE/g. Our results also demonstrated that the acetone extract of RBC1 treatment exhibits the lowest (33.75 mg CE/g), and FBC3 treatment exhibits the highest flavonoid concentrations (180.49 mg CE/g) (Table 5). This pattern implies that in all growth stages, the amounts of flavonoids tend to rise as the treatment is changed (fermented > blanched > raw). The ethanol extracts showed fluctuations in flavonoid contents, but there was no clear pattern based on the processing technique (RBC, BBC and FBC) or development stage. The flavonoid content of the powdered leaves of *B. campestris* ranged from 393.75 to 432.24 mg CE/g when extracted with ethanol. On the other hand, the *n*-hexane extracts of *B. campestris* exhibited the highest flavonoid concentration (338.73 mg CE/g) in FBC1 treatment, while BBC2 treatment showed the second highest flavonoid concentration of 327.01 mg CE/g. In contrast, it was noticed that the methanol extract of the FBC3 treatment showed the highest flavonoid content (448.17 mg CE/g), whereas the RBC2 treatment exhibited the lowest flavonoid content (236.35 mg CE/g). The pattern of higher flavonoid extractants in FBC, followed by BBC and RBC, emphasizes the role of both the growth stage and treatments in determining flavonoid accumulation.

The free radical scavenging ability of *B. campestris* was evaluated using various solvents, including *n*-hexane, acetone, ethanol and methanol. Findings showed that fermentation, among treatments, and acetone and methanol, among extractions, produced samples with strong antioxidant activity. The antioxidant activity of ethanol-extracted samples was typically moderate, while the *n*-hexane-extracted samples often exhibited moderate to high antioxidant activity. As for the treatments, our findings showed that FBC2 among the fermentation treatments exhibits the highest DPPH radical scavenging activity (% inhibition 71.24), while BBC3 displayed the lowest antioxidant activity (% inhibition 34.45) among the blanched samples in comparison with the other treated samples extracted with acetone (Table 5). The FBC2 treatment extracted with ethanol showed considerably higher antioxidant activity (% inhibition 49.77) and BBC3 the lowest (29.56%) among the growth stages. Similarly, the RBC2 treatment displayed the highest DPPH radical scavenging activity (77.06%) and BBC3 the lowest (35.57%) among the treatments when extracted with *n*-hexane. Moreover, the DPPH radical scavenging activity was more pronounced in methanol-extracted samples as RBC3 was shown to be one of the most effective samples in this solvent group, exhibiting the highest radical scavenging activity (98.80%), followed by FBC2 (94.32%) and FBC1 (91.88%).

FRAP values were affected by growth stages, treatments and type of extraction solvents. RBC1 showed higher activity, followed by RBC3 and RBC2. Among treatments, the trend was recorded as raw, followed by fermented, and then blanched, but in methanol extraction, an inverse trend was observed as fermented, followed by blanched, then raw. Our results revealed that the acetone extract of RBC1 treatment exhibits the highest FRAP value (125.51 µg/mL), suggesting that among the growth stages and treatments, raw *B. campestris* leaves had the better antioxidant capacity. FBC3 treatment showed the highest FRAP value at 150.09 µg/mL in ethanol extracts, while FBC1 treatment showed the second highest FRAP value (134.81 µg/mL). The FRAP values of methanol extracts indicated that RBCI treatment exhibits a moderate FRAP value (98.07 µg/mL) and FBC3 the highest FRAP value (107.72 µg/mL), demonstrating that fermentation treatment increased the antioxidant capacity at this point of growth. In contrast, *n*-hexane extracts exhibited low to moderate activity. In summary, the trend in antioxidant capacity is more pronounced in extracts of ethanol and methanol and less pronounced in extracts of acetone and hexane.

### 3.7. Anti-Nutritional Factors Found in the B. campestris Leaves

Results from this study showed that *B. campestris* contains a notable amount of anti-nutrients (Table 6), including tannins, saponins, phytates, oxalates and alkaloids that may adversely affect nutrient bioavailability. Presented in Table 6 are anti-nutritional factors found in the *B. campestris* leaves. Results in Table 6 show that the growth stage and treatments (blanching and fermentation) have a positive impact on the nutrient inhibitors. In addition, our findings indicated that the highest phytates contents are found in RBC1 (134.70 mg/100 g), whereas treatments such as blanching and fermentation showed a positive impact, as manifested by the lowered levels in BBC1 (75.57 mg/100 g) and FBC1 (44.01 mg/100 g). Similarly, results revealed a notable decline in the *B. campestris* tannin contents subjected firstly to blanching and then fermentation. Blanching was observed to have a minor decline, but fermentation was found to have a major impact on the tannin reduction. At all growth stages, the oxalate and alkaloid contents of *B. campestris* increased with the growth stages and decreased because of blanching and fermentation treatments, illustrating the positive effects. On the other hand, saponins decreased as the plant matured, and further reductions were caused by blanching and then fermentation.

## 4. Discussion

The morphological parameters (Table 1) of *B. campestris* were recorded in the fourth, sixth and eighth weeks of plant growth, and the results indicated that the plants were most matured and developed during their eighth week of growth. The number of leaves per plant was also maximum during the eighth week, with a larger surface area for the absorption of additional light to produce more energy. Higher leaf area is a sign of a leaf having more photosynthesizing mesophyll tissues, which, in turn, affects the leaf’s potential for photosynthetic activity [41].

Findings from this study (Figure 1) indicated that as fermentation time increased, the pH of the samples dropped from 6.48 to 4.11, and the titratable acidity of the samples increased, ranging from 0.1 to 0.9. This could be because fermentation by-products, such as lactic acid or other organic acids, are responsible for their formation [42]. In general, the samples’ pH values decreased, and their acidity levels increased as the growth period of the plant as well as the fermentation duration increased. The breakdown of organic compounds, the creation of different metabolites and microbial activity may all be responsible for the pH drop and the rise in acidity over time. The pH and acidity of foods are important determinants of their flavor, texture and safety [43]. According to Ray and Panda [44], certain bacteria (*Lactobacillus* and *Streptococcus*) are acid-tolerant and may survive at low pH levels (3.0–4.0). The quick reduction in pH suggests that the acidity of fermenting samples increased due to the formation of lactic acid by lactic acid bacteria (LAB). These results agree with those obtained by Misci et al. [45], who fermented pumpkin leaves for 168 h and measured the decrease in pH value from 7.13 to 4.23. In this regard, vegetable fermentation is a method used to preserve vegetables; it allows leftover and surplus vegetables to be kept inexpensively and cost-effectively, preventing deterioration by microbes [46].

A plant with more chlorophyll content is believed to have a higher potential for photosynthetic activity and overall plant health. “Chlorophyll a” and “chlorophyll b” are the chlorophyll pigments that collectively make the total chlorophyll used in photosynthesis [47]. The differences in growth circumstances, nutritional availability and other aspects specific to each treatment could be the reason behind the variability in chlorophyll concentration between treatments and growth stages (Table 2). Along this line, Mulay and Kokate [48] reported lower levels of chlorophyll a and b contents in the adult leaves of the poisonous flowering plant *Datura stramonium* than the current study’s results. The dark green color powders, which were produced by temperature changes and other chemical reactions during the fermentation and blanching processes, helped to destroy the chlorophyll and alter the color [49]. Since chlorophyll a is the primary pigment, and chlorophyll b is an accessory pigment, the concentration of chlorophyll a was higher than that of chlorophyll b [20]. Różyło and coworkers [49] observed considerably lower values of a* (−13.7) for fresh spinach leaves compared to fresh stems (−10.2), indicating a significantly higher share of green color in the leaves. The current investigation found similar results of a* value for fresh leaves and powders of *B. campestris* leaves that were drastically reduced in the treated (blanched and fermented) powders. Moreover, James and colleagues [50] reported that the young leaves have maximum mesophyll content; due to that, they appear blue–gray in color, while the adult leaves were shown to have a low concentration of mesophyll because they appear green in color.

In the present study, the analysis of the proximate composition of *B. campestris* at at each stage was performed to evaluate the best consumption stage of *B. campestris* with a minimum load of toxicants, as well as their reduction with the help of blanching and fermentation treatments. The nutritional importance of *B. campestris* can be seen from its nutritional composition in comparison with the other species from the Brassica family. Within this context, Hossain et al. [24] and Abid et al. [51] showed higher amounts of moisture and ash contents in several kinds of mustard and rapeseed than the current study’s results (Table 3). Abid et al. [51] determined that the fat content of *B. rapa* was 3.48%, which is lower than our findings. Moreover, these researchers [51] found an 11.90% protein concentration in *B. rapa*, which was nearly identical to the current study’s obtained values for protein in *B. campestris*. They also found greater amounts of crude fiber content, up to 44.18%, in cabbage on a dry weight basis [51]. The higher fiber content serves a physiological function by maintaining internal distension for proper peristaltic movement of the intestinal system, ensuring that food moves smoothly through the digestive tract. On the other hand, Hossain and coworkers [24] found significantly lower carbohydrate levels (17.02%) in BARI Sarisha-13, but Abid et al. [51] found about identical amounts of carbohydrates (57.45%) in *B. rapa* to *B. campestris*. Rapeseed has a low amount of accessible energy due to its high fiber content, as revealed by Feng and Zuo [52]. In the present analysis, the gross energy contents were found to be between 370–380 kcal/100 g, which is considerably higher than cabbage (189.25 kcal/100 g) and comparable to spinach leaves (300.94 kcal/100 g) [51]. According to Abdi et al. [51], the gross energy content of carbohydrates is primarily polysaccharides that have anti-ulcer, laxative, hypoglycemic, immunomodulatory, analgesic, expectorant, hypocholesterolemic and anabolic properties. They help to reduce the toxicity of cytostatic medicines and antibiotics. The most important function is their influence on the pharmacodynamics of plant-derived medications [53].

Importantly, growth stages, blanching and fermentation notably influenced the proximate composition. Fermentation treatment increased the ash, protein and fiber contents, whereas moisture, fat and carbohydrate contents decreased among *B. campestris* samples, and the trend was similar for all three stages. In addition, the results of the present investigation agree with previous findings of [54,55,56]. A continued fermentation for a long time may result in decreased fat content that could be attributed to lipolytic activities and the utilization of lipids as an energy source by the fermenting microorganisms [57]. Fermented *B. campestris* samples showed increased protein content due to fermentation hydrolysis and the release of embryonic proteins for seed germination [58]. This agrees with the findings of Nimra and Adeela’s study, which found an increase in protein content due to fermentation. Protein is crucial for child development and growth, and plant protein digestibility increases through fermentation [59]. In contrast, carbohydrates are the main source of energy in the human body. Non-fermented *B. campestris* samples have a higher carbohydrate content when compared to the fermented samples. Reduction in carbohydrate content is due to the utilization of starch during the metabolic activities of the fermentative microorganism and results in lower concentrations [58].

Flavonoids are secondary metabolites found in plants; they are responsible for their flavor, color and aroma. Plant flavonoids can act as signal molecules, UV filters and ROS scavengers. They also play several functions in drought, heat and frost tolerance [60]. Results of the present investigation (Table 5) showed that blanching causes a decrease in total phenolic and flavonoids, whereas fermentation causes the opposite. Our results agree with results obtained by other researchers who found that fermentation leads to a slight increase in phytochemical contents [55,61]. Our findings indicated that the ethanol, *n*-hexane and methanol extracts have higher flavonoid concentrations in *B. campestris* as compared to the total flavonoid content (TFC) in the various vegetable samples ranging from 1.09 to 3.59 mg CE/g. In this respect, high TFC exhibits excellent antibacterial, anti-inflammatory, antiallergic, antineoplastic, antiviral, antithrombotic and vasodilator activity [62]. The redox characteristics of phenolic compounds allow them to act as reducing agents, hydrogen donors, singlet oxygen quenchers, heavy metal chelators and hydroxyl radical quenchers, which contribute to their antioxidant action. Furthermore, flavonoids are responsible for a variety of medicinal activities, including anticancer, anti-inflammatory, antiviral, antibacterial, and anti-allergy qualities [51].

DPPH free radical scavenging is a common technique for evaluating the antioxidant properties of plant extracts. In the DPPH assay, the addition of the extract causes a concentration-dependent conversion of the violet DPPH solution to the yellow by-product, diphenylpicryl hydrazine [3,63]. On the other hand, the FRAP assay measures the reducing potential of an antioxidant reacting with a ferric tripyridyltriazine (Fe^3+^-TPTZ) complex, producing a colored ferrous tripyridyltriazine (Fe^2+^-TPTZ). At a low pH of about 3.6, reduction of Fe^3+^-TPTZ complex to blue-colored Fe^2+^-TPTZ takes place, which has an absorbance at 593 nm [64]. Results of the current investigation (Table 5) revealed slightly higher RSA values (98.80%) than those determined by Chu et al. [65] and Shyamala et al. [66], who discovered that spinach had the greatest DPPH RSA (85%). The stable free radical scavenging potential and FRAP values of samples extracted with *n*-hexane, acetone and ethanol were in the order of raw, followed by fermented, and then blanched, but an inverse order was observed in the case of the methanol extract. Previously, the DPPH inhibition potential of fermented samples extracted with *n*-hexane and ethanol was slightly lower as compared to non-fermented samples, but a slight increase was observed in the case of water extraction [55]. In another investigation, fermentation caused a notable increase in DPPH assay wherein IC_50_ of fermented sample decreased from 6.3 to 2.9 mg/mL [61]. In this regard, vegetable-rich phenolic compounds play an important function in anti-inflammatory and anticarcinogenic disorders, as well as in the prevention of oxidative stress-related diseases [67]. Phenolic compounds are significant plant ingredients that have antioxidant action due to their redox characteristics. Plant extracts’ hydroxyl groups are responsible for enhancing free radical scavenging [27].

Findings showed that *B. campestris* has a notable amount of anti-nutrients, including tannins, saponins, phytates, oxalates and alkaloids that may adversely affect nutrient bioavailability. Treatments, including blanching and fermentation, caused a significant decline in the nutrient inhibitors, improved the proximate composition and did not impact the antioxidant potential. Phytates, also known as phytic acids, are typically included in plant-based diets and are regarded as “anti-nutrients” because they can hinder the absorption of vital minerals like calcium, zinc and iron by the digestive system [68]. Tannins are polyhydric phenols that are found in almost all plant components and are known to inhibit the activities of trypsin, chymotrypsin, amylase and lipase [69]. Saponins have a bitter taste, are toxic in high quantities and interfere with the body’s ability to absorb nutrients by interacting with minerals like iron, zinc and vitamin E and inhibiting certain metabolic processes [32]. Oxalate, also known as oxalic acid, can combine with various minerals, such as sodium, potassium, calcium, iron and magnesium, to produce insoluble salts. It has been proposed that plants produce oxalate for several purposes, including calcium management, plant defense and heavy metal detoxification [70].

Findings from this study (Table 6) showed that blanching caused 46, 34, 1, 15 and 10% reduction in phytates, saponin, tannin, oxalate, and alkaloid contents, respectively, and rendered minerals more accessible when compared to samples of raw *B. campestris* leaves. In this regard, our findings agree with those of Mosha et al. [23], who found that cabbage processing by blanching led to a significant reduction in phytates (73%) and tannins (88%). In another investigation, blanching treatment was reported to cause a reduction in phytates (73%), tannins (85%), oxalates (69%) and alkaloids (68%) in spinach powder [71]. Similarly, blanching treatment was reported to cause a significant decline in phytates, tannins, oxalates and alkaloids in cabbage powder at 82, 79, 69 and 68%, separately [72]. Blanching is considered as a reliable method to reduce anti-nutrients because of heat inactivation and leaching during the soaking [23]. Similarly, our findings revealed that fermentation caused a 42, 49, 10, 7 and 6% reduction in phytates, saponins, tannins, oxalates and alkaloids, respectively, in comparison with raw samples of *B. campestris* leaves. Fermentation is reported to reduce phytates because, in addition to bacterial phytase activity, lactic acid bacteria activate endogenous phytates by lowering the pH to 4–5 [73]. Reduction in saponin content after fermentation may be attributed to the action of β-glucosidase, which catalyzes the structural degradation of saponins, resulting in their removal from the plant matrix [74]. Our results agree with previous findings, which showed that fermentation treatment causes a significant decrease in phytates (78%), oxalates (72%) and alkaloids (66%). In addition, 86% and 89% reduction in phytates and tannin contents were also reported by fermentation treatment. Anti-nutrients may have a deleterious impact on human health by lowering the bioavailability of minerals and the digestion of proteins. Oxalate, tannin and phytate levels were high in *B. campestris*, but saponin levels were low. However, if adequately processed, the presence of modest quantities of these anti-nutrients should not be a concern because processing reduces the level of anti-nutrients to acceptable levels [75].

## 5. Conclusions

The results of this study concluded that *B. campestris* is one of the world’s most beloved green leafy vegetables, as it is primarily consumed in Asia due to its exceptional nutritional composition and health-promoting attributes. Additionally, it also contains a notable amount of anti-nutrients, i.e., phytates, tannins, alkaloids, oxalates and saponins, that are known to reduce nutrient accessibility and bioavailability due to their nutrient binding capacity. *B. campestris* nutritional profiling at three different growth stages unveiled that the second stage carries relatively lower levels of aforementioned toxicants and more nutrients as compared to the first and third stages. Blanching followed by fermentation improved the nutritional value, greatly decreased the anti-nutrient load, and had no effect on the phytochemical contents. Firstly, blanching almost caused a 46% reduction in phytates as compared to raw samples; secondly, fermentation of blanched samples caused a further reduction of 42% as compared to blanched samples. The combined approach of blanching followed by fermentation emerges as a promising strategy to enhance the nutritional quality of *B. campestris* while mitigating its anti-nutrient content. These findings advocate for the adoption of these food processing techniques to promote safer and more nutritious dietary choices. Future investigations could delve into additional factors, such as sensory attributes and consumer acceptance, to further validate the efficacy and acceptance of these methods in optimizing *B. campestris* as a beneficial dietary component.

## Figures and Tables

**Table 1:** Morphological attributes (mean ± standard deviation) of *B. campestris* harvested at different growth stages.

Plant Age (Week)	Root Size (inch)	Stem Size (inch)	Leaf Size (inch)	Leaf Width (inch)	Plant Height (inch)	Leaf Count
4th	7.22 ± 0.81 ^c^	12.10 ± 3.12 ^c^	9.17 ± 2.60 ^b^	3.80 ± 0.76 ^b^	19.32 ± 3.43 ^c^	9.00 ± 1.58 ^c^
6th	10.04 ± 1.36 ^b^	45.79 ± 8.03 ^b^	10.71 ± 2.69 ^b^	4.58 ± 3.48 ^ab^	55.83 ± 8.06 ^b^	45.00 ± 6.20 ^b^
8th	11.09 ± 2.20 ^a^	74.14 ± 7.59 ^a^	15.64 ± 3.25 ^a^	5.95 ± 3.66 ^a^	85.23 ± 8.03 ^a^	62.00 ± 5.10 ^a^
F-ratio	52.7 **	708 **	44.9 **	4.36 *	747 **	164 **

Note: Different lowercase superscript letters in the same column of the table show statistically different means (*p* < 0.05); letters a–c represent lettering for significant parameter results; * represent level of significance (*p* < 0.05); ** represent level of significance (*p* < 0.01).

**Table 2:** Chlorophyll a and b, total chlorophyll and anthocyanin contents (mean ± standard deviation) in *B. campestris* leaves.

Samples	Chlorophyll a	Chlorophyll b	Total Chlorophyll	Anthocyanin (mg/g)
RBC1	31.98 ± 0.06 ^c^	51.82 ± 0.11 ^ab^	83.80 ± 0.09 ^bc^	134.33 ± 4.53 ^bc^
BBC1	31.75 ± 0.05 ^c^	51.46 ± 0.07 ^ab^	83.21 ± 0.11 ^bc^	131.74 ± 5.98 ^bc^
FBC1	32.81 ± 0.03 ^a^	52.04 ± 0.08 ^a^	84.85 ± 0.11 ^ab^	124.02 ± 4.33 ^c^
RBC2	32.27 ± 0.48 ^b^	49.59 ± 3.92 ^bc^	81.85 ± 3.44 ^cd^	159.50 ± 8.87 ^a^
BBC2	31.70 ± 0.04 ^c^	51.28 ± 0.03 ^ab^	82.98 ± 0.07 ^bc^	138.52 ± 7.63 ^b^
FBC2	32.99 ± 0.02 ^a^	52.87 ± 0.04 ^a^	85.86 ± 0.04 ^a^	136.29 ± 8.30 ^b^
RBC3	32.93 ± 0.02 ^a^	39.16 ± 0.02 ^e^	72.08 ± 0.04 ^f^	158.85 ± 4.04 ^a^
BBC3	32.32 ± 0.04 ^b^	44.72 ± 0.01 ^d^	77.04 ± 0.03 ^e^	156.28 ± 5.03 ^a^
FBC3	33.06 ± 0.07 ^a^	47.69 ± 0.03 ^c^	80.75 ± 0.08 ^d^	135.25 ± 5.91 ^b^
F-ratio	31.4 **	35.2 **	42.4 **	12.9 **

Note: Different lowercase superscript letters in the same column of the table show statistically different means (*p* < 0.05); letters a–f represent lettering for significant parameter results; ** represent level of significance (*p* < 0.01); RBC1: first stage of *B. campestris* raw leaf powder; BBC1: first stage of *B. campestris* blanched leaf powder; FBC1: first stage of *B. campestris* fermented leaf powder; RBC2: second stage of *B. campestris* raw leaf powder; BBC2: second stage of *B. campestris* blanched leaf powder; FBC2: second stage of *B. campestris* fermented leaf powder; RBC3: third stage of *B. campestris* raw leaf powder; BBC3: third stage of *B. campestris* blanched leaf powder; FBC3: third stage of *B. campestris* fermented leaf powder.

**Table 3:** Proximate composition (mean ± standard deviation) of *B. campestris* harvested at different growth stages.

Plant Part	Moisture (%)	Total Ash (%)	Crude Fat (%)	Crude Protein (%)	Crude Fiber (%)	NFE (%)	Energy (Kcal/100 g)
RBC1	0.91 ± 0.06 ^bc^	5.36 ± 0.16 ^b^	9.88 ± 1.05 ^cd^	9.02 ± 0.62 ^d^	9.58 ± 0.15	64.47 ± 0.79 ^a^	381.07 ± 6.66 ^abc^
BBC1	0.91 ± 0.07 ^bc^	5.25 ± 0.22 ^b^	9.46 ± 0.05 ^bcd^	9.48 ± 1.21 ^d^	9.36 ± 0.64	64.48 ± 1.39 ^a^	385.91 ± 3.11 ^a^
FBC1	0.82 ± 0.06 ^c^	5.98 ± 0.50 ^b^	9.16 ± 0.52 ^cd^	10.43 ± 0.47 ^cd^	10.71 ± 0.07	64.25 ± 0.37 ^a^	382.21 ± 1.18 ^ab^
RBC2	1.05 ± 0.09 ^b^	5.58 ± 0.19 ^b^	9.36 ± 0.08 ^d^	11.03 ± 1.66 ^bcd^	9.23 ± 0.39	61.66 ± 2.40 ^ab^	370.62 ± 1.43 ^c^
BBC2	0.91 ± 0.10 ^bc^	5.58 ± 0.02 ^b^	10.10 ± 0.06 ^bc^	11.93 ± 1.95 ^bc^	10.07 ± 2.47	61.41 ± 4.41 ^ab^	383.88 ± 10.28 ^a^
FBC2	0.77 ± 0.03 ^c^	7.87 ± 0.81 ^a^	9.00 ± 0.21 ^b^	14.22 ± 1.17 ^a^	9.77 ± 2.08	59.12 ± 1.65 ^bc^	387.19 ± 8.80 ^a^
RBC3	1.37 ± 0.16 ^a^	8.12 ± 0.68 ^a^	9.64 ± 0.14 ^cd^	8.85 ± 0.79 ^d^	9.32 ± 0.98	63.17 ± 1.35 ^a^	372.56 ± 6.92 ^bc^
BBC3	1.01 ± 0.10 ^b^	5.57 ± 0.07 ^b^	12.01 ± 0.51 ^a^	11.85 ± 1.29 ^c^	9.19 ± 1.20	57.46 ± 1.79 ^c^	385.34 ± 4.69 ^a^
FBC3	0.79 ± 0.03 ^c^	8.52 ± 0.43 ^a^	9.11 ± 0.37 ^cd^	12.81 ± 1.85 ^ab^	9.93 ± 0.89	60.99 ± 2.43 ^abc^	382.40 ± 5.79 ^ab^
F-ratio	12.3 **	46.2 **	12.3 **	4.62 **	0.47 ^ns^	3.10 *	2.66 *

Note: Different lowercase superscript letters in the same column of the table show statistically different means (*p* < 0.05); letters a–d represent lettering for significant parameter results; * represent level of significance (*p* < 0.05); ** represent level of significance (*p* < 0.01); ^ns^ represent level of non-significance (*p* > 0.05); RBC1: first stage of *B. campestris* raw leaf powder; BBC1: first stage of *B. campestris* blanched leaf powder; FBC1: first stage of *B. campestris* fermented leaf powder; RBC2: second stage of *B. campestris* raw leaf powder; BBC2: second stage of *B. campestris* blanched leaf powder; FBC2: second stage of *B. campestris* fermented leaf powder; RBC3: third stage of *B. campestris* raw leaf powder; BBC3: third stage of *B. campestris* blanched leaf powder; FBC3: third stage of *B. campestris* fermented leaf powder.

**Table 4:** Color tonality (mean ± standard deviation) of *B. campestris* leaves.

Treatment	L*	a*	b*
FrBC1	54.80 ± 1.02 ^e^	–9.72 ± 1.29 ^g^	21.37 ± 3.08 ^f^
RBC1	60.69 ± 1.33 ^bc^	–9.29 ± 0.16 ^fg^	34.14 ± 0.76 ^b^
BBC1	57.17 ± 0.31 ^d^	–6.72 ± 0.39 ^e^	29.30 ± 0.70 ^c^
FBC1	56.68 ± 0.49 ^d^	–0.89 ± 0.29 ^b^	24.94 ± 0.98 ^de^
FrBC2	53.52 ± 1.78 ^f^	–8.35 ± 0.98 ^f^	16.36 ± 0.54 ^h^
RBC2	59.26 ± 2.14 ^c^	−1.23 ± 0.62 ^b^	36.06 ± 0.43 ^b^
BBC2	59.14 ± 1.01 ^c^	–4.60 ± 0.60 ^d^	34.36 ± 1.34 ^b^
FBC2	55.39 ± 0.94 ^de^	–0.92 ± 0.10 ^b^	23.53 ± 1.10 ^e^
FrBC3	54.16 ± 1.32 ^ef^	–9.04 ± 0.88 ^fg^	18.87 ± 1.81 ^g^
RBC3	66.65 ± 0.89 ^a^	–4.25 ± 0.57 ^d^	38.90 ± 1.05 ^a^
BBC3	62.17 ± 0.44 ^b^	–2.53 ± 0.05 ^c^	33.91 ± 1.22 ^b^
FBC3	51.88 ± 1.22 ^g^	2.63 ± 0.21 ^a^	26.55 ± 1.96 ^d^
F-ratio	48.0 **	131 **	109 **

Note: Different lowercase superscript letters in the same column of the table show statistically different means (*p* < 0.05); letters a–h represent lettering for significant parameter results; ** represent level of significance (*p* < 0.01); RBC1: first stage of *B. campestris* raw leaf powder; BBC1: first stage of *B. campestris* blanched leaf powder; FBC1: first stage of *B. campestris* fermented leaf powder; RBC2: second stage of *B. campestris* raw leaf powder; BBC2: second stage of *B. campestris* blanched leaf powder; FBC2: second stage of *B. campestris* fermented leaf powder; RBC3: third stage of *B. campestris* raw leaf powder; BBC3: third stage of *B. campestris* blanched leaf powder; FBC3: third stage of *B. campestris* fermented leaf powder.

**Table 5:** Antioxidant potential (mean ± standard deviation) of the *B. campestris* leaf powder extracts.

Solvent	Sample	Acetone	Ethanol	*n*-Hexane	Methanol
DPPH (%)	RBC1	83.22 ± 3.11 ^a^	37.71 ± 0.82 ^b^	62.11 ± 0.67 ^c^	61.86 ± 0.03 ^g^
	BBC1	37.27 ± 2.28 ^de^	34.29 ± 0.87 ^cd^	42.03 ± 0.34 ^f^	85.66 ± 0.02 ^e^
	FBC1	39.32 ± 3.16 ^d^	37.46 ± 0.21 ^b^	43.46 ± 0.25 ^ef^	91.88 ± 0.08 ^c^
	RBC2	84.82 ± 3.73 ^a^	35.63 ± 0.47 ^bc^	77.06 ± 2.46 ^a^	59.15 ± 0.04 ^h^
	BBC2	37.70 ± 4.27 ^de^	32.63 ± 0.24 ^de^	39.21 ± 0.47 ^g^	62.05 ± 0.01 ^g^
	FBC2	51.09 ± 1.89 ^c^	49.77 ± 2.64 ^a^	50.48 ± 0.61 ^d^	94.32 ± 0.02 ^b^
	RBC3	86.87 ± 1.30 ^a^	30.83 ± 0.29 ^ef^	75.20 ± 0.55 ^b^	98.80 ± 0.80 ^a^
	BBC3	34.45 ± 0.05 ^e^	29.56 ± 0.56 ^f^	35.57 ± 0.55 ^h^	67.01 ± 0.08 ^f^
	FBC3	71.24 ± 1.06 ^b^	34.45 ± 0.36 ^cd^	43.81 ± 0.37 ^e^	88.61 ± 0.06 ^d^
	F-ratio	222 **	56.2 **	820 **	10,604 **
TPC (mg GAE/g)	RBC1	140.30 ± 10.02 ^de^	357.99 ± 9.33 ^c^	341.36 ± 7.52 ^b^	306.17 ± 0.16 ^d^
	BBC1	186.13 ± 9.83 ^b^	363.93 ± 1.01 ^b^	93.88 ± 4.53^1^	361.53 ± 10.81 ^b^
	FBC1	245.59 ± 6.04 ^a^	388.28 ± 2.14 ^a^	203.39 ± 7.55 ^g^	377.91 ± 12.17 ^a^
	RBC2	124.40 ± 5.50 ^e^	338.82 ± 1.02 ^d^	178.85 ± 9.78 ^h^	279.66 ± 12.03 ^e^
	BBC2	155.39 ± 7.35 ^d^	370.37 ± 1.33 ^a^	302.96 ± 3.91 ^d^	287.22 ± 2.41 ^e^
	FBC2	246.78 ± 5.31 ^a^	384.78 ± 1.14 ^b^	367.71 ± 9.42 ^a^	387.35 ± 7.96 ^a^
	RBC3	153.57 ± 9.30 ^f^	375.24 ± 3.24 ^b^	316.82 ± 10.09 ^c^	345.16 ± 9.38 ^c^
	BBC3	77.40 ± 4.98 ^g^	339.54 ± 0.87 ^c^	270.29 ± 3.98 ^f^	352.45 ± 5.97 ^b^
	FBC3	171.22 ± 9.87 ^c^	356.54 ± 5.68 ^d^	283.43 ± 6.53 ^e^	360.84 ± 5.14 ^b^
	F-ratio	145 **	71.8 **	419 **	67.5 **
FRAP (µg/mL)	RBCI	125.51 ± 0.18 ^a^	101.48 ± 1.95 ^f^	77.87 ± 1.19 ^bc^	98.07 ± 1.89 ^b^
	BBC1	87.65 ± 1.32 ^c^	119.67 ± 0.13 ^c^	75.09 ± 0.44 ^d^	66.53 ± 0.44 ^f^
	FBC1	108.23 ± 0.13 ^b^	134.81 ± 0.36 ^b^	78.59 ± 0.42 ^b^	89.20 ± 1.42 ^d^
	RBC2	88.29 ± 5.39 ^c^	110.21 ± 1.06 ^d^	73.75 ± 0.38 ^e^	57.69 ± 0.15 ^g^
	BBC2	85.79 ± 2.01 ^c^	96.12 ± 1.02 ^g^	77.15 ± 0.38 ^c^	56.58 ± 0.36 ^g^
	FBC2	86.47 ± 1.17 ^c^	105.12 ± 0.26 ^e^	74.23 ± 0.14 ^de^	75.47 ± 0.24 ^e^
	RBC3	86.60 ± 1.68 ^c^	101.61 ± 1.14 ^f^	74.42 ± 0.44 ^de^	90.82 ± 0.42 ^c^
	BBC3	73.42 ± 0.53 ^e^	101.15 ± 1.97 ^f^	77.10 ± 0.51 ^c^	88.40 ± 0.17 ^d^
	FBC3	78.22 ± 0.94 ^d^	150.09 ± 0.15 ^a^	80.43 ± 1.64 ^a^	107.72 ± 0.13 ^a^
	F-ratio	172 **	784 **	26.8 **	1412 **
Flavonoids (mg CE/g)	RBC1	33.75 ± 2.81 ^g^	395.32 ± 7.87 ^b^	167.42 ± 7.58 ^d^	386.01 ± 8.33 ^d^
	BBC1	93.04 ± 4.74 ^cd^	422.42 ± 7.91 ^a^	214.77 ± 6.56 ^c^	414.02 ± 6.31 ^c^
	FBC1	148.61 ± 3.12 ^b^	432.24 ± 12.71 ^a^	338.73 ± 2.46 ^a^	430.73 ± 8.20 ^b^
	RBC2	41.52 ± 1.46 ^f^	399.88 ± 7.36 ^b^	71.31 ± 3.07 ^e^	236.35 ± 2.60 ^e^
	BBC2	89.00 ± 3.81 ^d^	403.06 ± 3.44 ^b^	284.99 ± 3.59 ^b^	380.95 ± 4.56 ^d^
	FBC2	142.70 ± 5.82 ^b^	404.47 ± 2.84 ^b^	327.01 ±2.77 ^a^	409.72 ± 5.63 ^c^
	RBC3	78.49 ± 1.51 ^e^	402.10 ± 3.17 ^b^	81.27 ± 1.56 ^e^	406.68 ± 2.27 ^c^
	BBC3	97.50 ± 7.21 ^c^	395.27 ± 0.47 ^b^	175.31 ± 8.13 ^d^	411.23 ± 3.54 ^c^
	FBC3	180.49 ± 3.90 ^a^	393.75 ± 9.19 ^b^	221.47 ± 2.32 ^c^	448.17 ± 4.43 ^a^
	F-ratio	1826 **	10.4 **	460 **	376 **

Note: Different lowercase superscript letters in the same column of the table show statistically different means (*p* < 0.05); letters a–h represent lettering for significant parameter results; ** represent level of significance (*p* < 0.01); RBC1: first stage of *B. campestris* raw leaf powder; BBC1: first stage of *B. campestris* blanched leaf powder; FBC1: first stage of *B. campestris* fermented leaf powder; RBC2: second stage of *B. campestris* raw leaf powder; BBC2: second stage of *B. campestris* blanched leaf powder; FBC2: second stage of *B. campestris* fermented leaf powder; RBC3: third stage of *B. campestris* raw leaf powder; BBC3: third stage of *B. campestris* blanched leaf powder; FBC3: third stage of *B. campestris* fermented leaf powder.

**Table 6:** Anti-nutritional factors (mean ± standard deviation) found in the *B. campestris* leaves.

Sample	Phytates (mg/100 g)	Tannins (mg/100 g)	Saponins (mg/100 g)	Oxalates (mg/100 g)	Alkaloids (mg/100 g)
RBC1	134.70 ± 0.20 ^a^	163.82 ± 4.75 ^ab^	30.85 ± 0.38 ^a^	1440.36 ± 47.91 ^c^	0.43 ± 0.07 ^cde^
BBC1	75.57 ± 0.29 ^c^	162.57 ± 2.86 ^abc^	20.26 ± 3.12 ^c^	1234.86 ± 15.85 ^de^	0.39 ± 0.02 ^def^
FBC1	44.01 ± 6.35 ^e^	147.70 ± 5.00 ^d^	11.98 ± 0.10 ^ef^	1155.14 ± 50.39 ^e^	0.37 ± 0.03 ^ef^
RBC2	28.67 ± 0.12 ^g^	160.32 ± 7.23 ^bc^	27.88 ± 0.37 ^ef^	1819.37 ± 55.84 ^a^	0.46 ± 0.05 ^c^
BBC2	26.46 ± 0.04 ^f^	154.24 ± 2.35 ^a^	12.68 ± 0.15 ^e^	1500.45 ± 46.05 ^c^	0.37 ± 0.05 ^def^
FBC2	19.21 ± 0.07 ^h^	57.36 ± 3.28 ^e^	13.39 ± 0.25 ^de^	1186.04 ± 71.54 ^e^	0.35 ± 0.03 ^f^
RBC3	87.04 ± 0.06 ^b^	168.92 ± 0.80 ^a^	14.98 ± 0.95 ^d^	1705.95 ± 51.93 ^b^	0.66 ± 0.03 ^a^
BBC3	66.48 ± 0.39 ^d^	158.02 ± 3.12 ^bc^	9.91 ± 1.80 ^f^	1278.38 ± 40.25 ^d^	0.56 ± 0.01 ^b^
FBC3	43.54 ± 0.05 ^e^	156.53 ± 4.94 ^c^	11.37 ± 0.96 ^ef^	1283.51 ± 51.61 ^d^	0.44 ± 0.04 ^cd^
F-ratio	845 **	209 **	103 **	66.6 **	19.8 **

Note: Different lowercase superscript letters in the same column of the table show statistically different means (*p* < 0.05); letters a–f represent lettering for significant parameter results; ** represent level of significance (*p* < 0.01); RBC1: first stage of *B. campestris* raw leaf powder; BBC1: first stage of *B. campestris* blanched leaf powder; FBC1: first stage of *B. campestris* fermented leaf powder; RBC2: second stage of *B. campestris* raw leaf powder; BBC2: second stage of *B. campestris* blanched leaf powder; FBC2: second stage of *B. campestris* fermented leaf powder; RBC3: third stage of *B. campestris* raw leaf powder; BBC3: third stage of *B. campestris* blanched leaf powder; FBC3: third stage of *B. campestris* fermented leaf powder.

## Data Availability

The original contributions presented in the study are included in the article, further inquiries can be directed to the corresponding authors.
